# Factors Associated with COVID-19 Vaccination Promptness after Eligibility in a North Carolina Longitudinal Cohort Study

**DOI:** 10.3390/vaccines11111639

**Published:** 2023-10-26

**Authors:** Coralei E. Neighbors, Richard A. Faldowski, Carl F. Pieper, Joshua Taylor, Megan Gaines, Richard Sloane, Douglas Wixted, Christopher W. Woods, L. Kristin Newby

**Affiliations:** 1 Department of Population Health, Duke University, Durham, NC 27701, USA; 2 Hubert-Yeargan Center for Global Health, Duke University, Durham, NC 27710, USA; 3 Center for the Study of Aging and Human Development, Duke University Medical Center, Durham, NC 27710, USA; 4 Department of Biostatistics and Bioinformatics, Duke University Medical Center, Durham, NC 27710, USA; 5 Duke Clinical and Translational Science Institute, Duke University, Durham, NC 27701, USAkristin.newby@duke.edu (L.K.N.); 6 Departments of Medicine and Pathology, Duke University Medical Center, Durham, NC 27710, USA; 7 Duke Clinical Research Institute, Duke University, Durham, NC 27701, USA; 8 Division of Cardiology, Department of Medicine, Duke University Medical Center, Durham, NC 27710, USA

**Keywords:** COVID-19, vaccination, vaccination promptness, vaccination intention, vaccination hesitancy, public health, North Carolina

## Abstract

Many studies identified factors associated with vaccination intention and hesitancy, but factors associated with vaccination promptness and the effect of vaccination intention on vaccination promptness are unknown. This study identified factors associated with COVID-19 vaccination promptness and evaluated the role of vaccination intention on vaccination promptness in 1223 participants in a community-based longitudinal cohort study (June 2020 to December 2021). Participants answered questions regarding COVID-19 vaccination intention, vaccination status, and reasons for not receiving a vaccine. The association of baseline vaccine hesitancy with vaccination was assessed by the Kaplan–Meier survival analysis. Follow-up analyses tested the importance of other variables predicting vaccination using the Cox proportional hazards model. Older age was associated with shorter time to vaccination (HR = 1.76 [1.37–2.25] 85-year-old versus 65-year-old). Lower education levels (HR = 0.80 [0.69–0.92]), household incomes (HR = 0.84 [0.72–0.98]), and baseline vaccination intention of ‘No’ (HR = 0.16 [0.11–0.23]) were associated with longer times to vaccination. The most common reasons for not being vaccinated (N = 58) were vaccine safety concerns (n = 33), side effects (n = 28), and vaccine effectiveness (n = 25). Vaccination campaigns that target populations prone to hesitancy and address vaccine safety and effectiveness could be helpful in future vaccination rollouts.

## 1. Introduction

Since the first Coronavirus disease 2019 (COVID-19) case in December 2019, the pandemic has had catastrophic effects on global health [1,2,3]. As of March 2023, over 100 million confirmed COVID-19 cases in the United States have resulted in over 1 million deaths, with approximately 13.3% of COVID-19 patients experiencing a post-COVID-19 condition one month or longer after infection [4,5]. North Carolina has over 3.4 million confirmed COVID-19 cases and nearly 29,000 confirmed deaths [6]. Due to these devastating effects, solutions to stop the spread of COVID-19 were rapidly developed. Vaccination is the most cost-efficient method of avoiding infectious diseases or preventing severe consequences of infection and has been one of the most effective public health interventions for COVID-19 [7,8,9,10,11,12,13,14].

COVID-19 vaccines were first distributed in the United States in December 2020, with the first North Carolinians receiving a vaccine on 14 December 2020 [15,16]. Currently, three main types of COVID-19 vaccines have been approved or authorized for use in the United States: mRNA vaccines (Pfizer-BioNTech and Moderna), protein subunit vaccines (Novavax), and viral vector vaccines (Johnson & Johnson’s Janssen) [17]. Despite evidence of COVID-19 vaccine efficacy, vaccination hesitancy continues to impede the goal of herd immunity and has been recognized as a threat to immunization programs worldwide [18,19,20].

As of 11 October 2021, it was estimated that 11.4% of North Carolinians planned to ‘probably not or definitely not’ receive the COVID-19 vaccine, with an additional 3.4% unsure if they would be willing to receive it [21]. As of May 2023, 69.5% of Americans and 63.0% of North Carolinians had completed their primary vaccination series against COVID-19 [22,23]. Current vaccination hesitancy and intention research highlights multiple influential factors in various populations [18,24,25]. These factors include the perceived risks of adverse side effects that the vaccines pose; the speed with which the COVID-19 vaccine was developed; medical mistrust and mistrust of the government, public health figures/organizations, and corporations; and the beliefs that COVID-19 is harmless, and vaccination is unnecessary [18,24,25,26,27,28,29,30,31,32,33,34,35,36]. Vaccination hesitancy has been associated with sociodemographic characteristics, such as sex, age, ethnicity, race, education level, income level, marital status, residence, political ideology, and previous vaccination history [25,27,31,32,37,38,39,40,41,42]. In North Carolina specifically, vaccination hesitancy has been found to be associated with being female or Black, government distrust, and safety concerns [43]. While many studies report on the factors associated with vaccination intention and vaccination hesitancy, the factors associated with vaccination promptness—how rapidly one receives vaccination after becoming eligible; the antithesis of vaccination hesitancy—and the role of vaccination intention in vaccination promptness have not yet been explored. This study aimed to (1) identify key factors associated with COVID-19 vaccination promptness and (2) evaluate the role of early (prior to vaccine availability) vaccination intention in observed COVID-19 vaccination promptness.

## 2. Materials and Methods

### 2.1. Study Sample

Data for this study were drawn from the Cabarrus County COVID-19 Prevalence and Immunity (C3PI) Study, a 1410-participant, community-based, longitudinal COVID-19 surveillance study. The complete design, methods, and baseline characteristics of the C3PI Study were published previously [44]. In brief, the C3PI Study enrolled participants from the Measurement to Understand the Reclassification of Disease of Cabarrus/Kannapolis (MURDOCK) Study Community Registry and Biorepository longitudinal cohort between June 2020 and August 2020 [45,46]. Once enrolled, participants were asked to complete a baseline survey and biweekly surveys for up to a total follow-up of 74 weeks (48 weeks in Phase 1 (June 2020 to April 2021) and 26 weeks in Phase 2 (May 2021 to December 2021)). Included in the surveys were questions regarding demographic, socioeconomic, employment, and clinic data, as well as pandemic-specific data, including use of mitigation behaviors, participant perceptions, intent to receive COVID-19 vaccination when a vaccine became available, and vaccination data (date/s of initial vaccination/vaccination series and any boosters and type of vaccine administered). During Phase 2, additional survey questions were added, asking participants their reasoning for not receiving a COVID-19 vaccine if they had not been vaccinated before May 2021.

**Figure 1:**
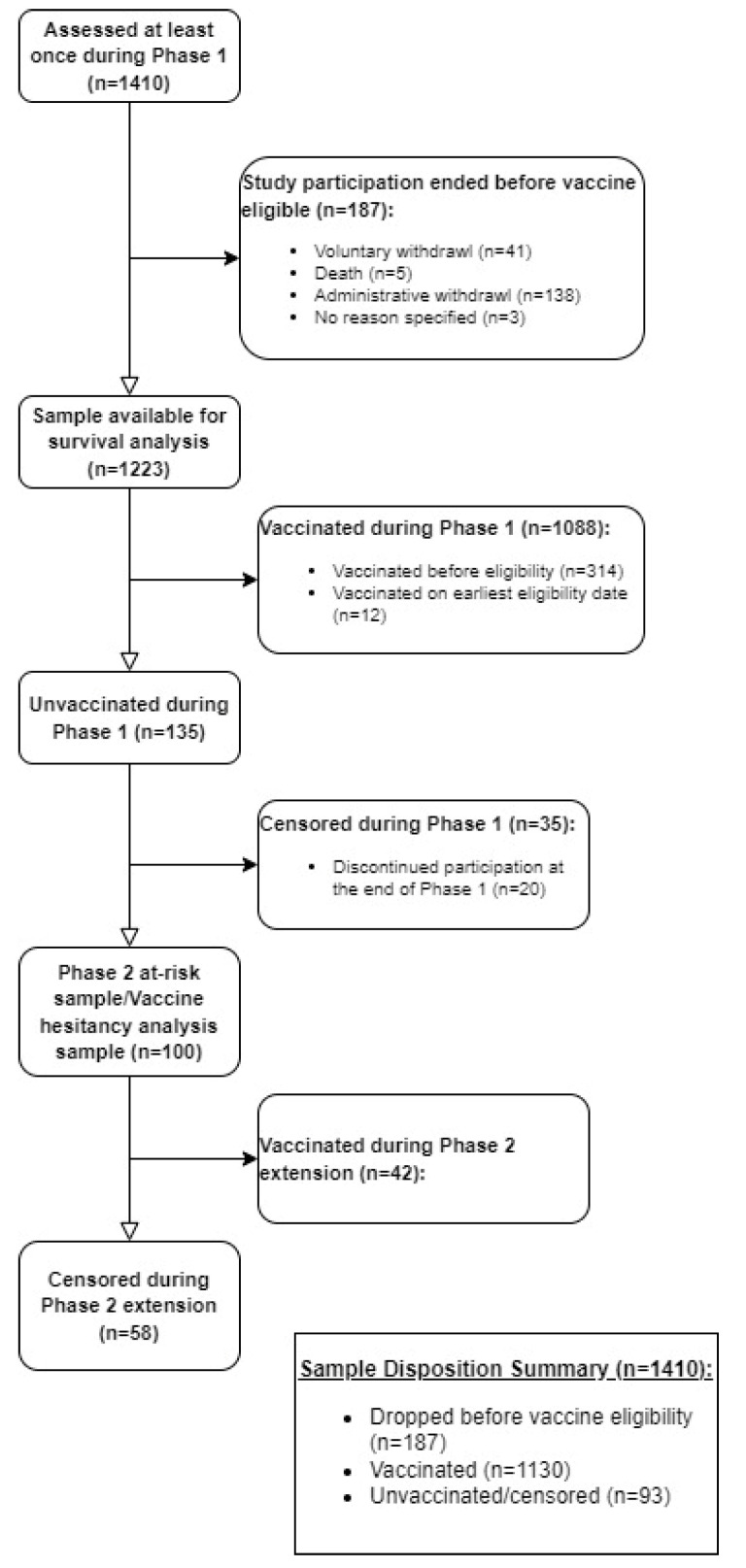
CONSORT diagram.

Figure 1 describes the study population. A total of 187 (13.3%) of 1410 potential participants were excluded from this analysis because, prior to vaccine availability or eligibility to receive a vaccine, they (i) died (n = 5, 2.7%), (ii) chose to withdraw from the study (n = 41, 21.9%), or (iii) were administratively withdrawn from the study after failing to complete ≥4 surveys (n = 138, 73.8%). The final analysis included 1223 participants. At the end of Phase 1, 1088 participants (89.0%) were vaccinated, and 135 (11.0%) participants were unvaccinated. Of the 135 unvaccinated participants, 15 (11.1%) participants were unvaccinated at the time they withdrew from the study during Phase 1 and were censored, 20 (14.8%) participants completed Phase 1 unvaccinated but declined to participate in Phase 2, and 100 (74.1%) were followed into Phase 2. A total of 42 (42.0%) were ultimately vaccinated and 58 (58.0%) remained unvaccinated when they left the study (censored). Thus, of the 1223 total participants included in the analysis, 1130 (92.4%) were vaccinated and 93 (7.6%) were censored and remained unvaccinated at last contact.

### 2.2. Study Measures and Definitions

#### 2.2.1. Vaccination Intention

For this study, vaccination intention was defined as a participant’s declared intention to receive a vaccination. Participants self-reported their intention of ‘Yes’, ‘No’, or ‘Unsure’ by asking, “Do you plan to get a vaccine for COVID-19 when one becomes available?”. This question was asked on the baseline and biweekly surveys until the participant received their COVID-19 vaccination. For modeling purposes, only the baseline responses were used.

#### 2.2.2. Vaccination Eligibility Date

Key sociodemographic and medical information obtained from the baseline and Phase 1 biweekly follow-up surveys were used to assign the North Carolina Department of Health and Human Services (NCDHHS) vaccination priority grouping to participants, including age, medical conditions, employment status, and occupation. After determining eligibility for all NCDHHS vaccination priority groups, the earliest date of vaccination eligibility was assigned to participants based on their highest priority grouping (Table 1). Priority groups 2 (43.4%) and 4a (23.6%) had the most participants, followed by priority groups 1 (11.9%) and 3b (11.5%).

#### 2.2.3. Vaccination Promptness

Vaccination promptness was defined as how rapidly participants received COVID-19 vaccinations after becoming eligible (in days) based on their earliest vaccination eligibility date. Self-reported vaccination status and dates were obtained from Phase 1 and 2 follow-up surveys. For 5 participants who had received a COVID-19 vaccine and were missing a vaccination date, survey completion dates were used as approximations for vaccination dates because survey dates generally differed from vaccination dates by no more than a few weeks among individuals with both.

#### 2.2.4. Vaccination Hesitancy

Vaccination hesitancy was measured by a set of questions about reasons for not receiving a COVID-19 vaccine. This question was asked to all participants who had not received a vaccine by the start of Phase 2 (May 2021, after all adults became eligible to receive a vaccine) and was asked for each Phase 2 follow-up survey until participants became vaccinated, withdrew from the study, or the study terminated. Participants could choose multiple options from the following ten choices: (1) I have not been able to schedule an appointment yet, (2) I am concerned about vaccine safety, (3) I am concerned about vaccine effectiveness, (4) I am afraid of needles, (5) I am concerned about side effects from the vaccination, (6) I am concerned based on the experience of a friend or family member, (7) I feel it would be hard for me due to transportation, other costs or burden on me, (8) I am not concerned about getting infected and becoming ill with COVID-19, (9) Religious or other exemption, and (10) Other, please specify. If a participant chose the “Other, please specify” option, they were asked to specify their reason in a free-text field.

### 2.3. Statistical Analysis

Vaccination promptness was modeled using survival analyses, which not only modeled time to vaccination but also accounted for participants who dropped out or were removed from the study early (censoring). In survival analyses, vaccination promptness was operationalized as the time to vaccination indexed by the difference between a participant’s earliest eligibility date and their vaccination date or censoring date +1 (to account for participants vaccinated on their eligibility date). Individuals vaccinated before their earliest eligibility dates were retained in survival analyses by assigning at-risk times of 0.5 days.

Initially, the bivariate association of potential predictors (such as sociodemographic characteristics, vaccination intention, and presence of clinical conditions) with time to vaccination was assessed using Kaplan–Meier survival analyses. Statistical significance was determined by the log-rank test at a type-I error rate of 0.05. Kaplan–Meier results were used to screen plausible predictors and evaluate whether multi-category categorical or ordinal covariates could be collapsed into smaller sets of internally homogeneous scores.

The strongest set of predictors, as defined by *p*-values less than 0.05 from the bivariate Kaplan–Meier analyses, were then incorporated into a multivariable Cox proportional hazards model. Interactions between baseline vaccination intention and other covariates were also considered. The proportional hazards assumption was examined for each predictor graphically and by testing for interactions between each covariate and log survival time [47,48]. The adequacy of the functional form of age in the model was examined with Martingale residuals [48]. All statistical tests were conducted at a 0.05 level of significance. All analyses were performed using SAS software version 9.4 (SAS Institute Inc., Cary, NC, USA).

#### Missing Data

In analyses, missing covariate values were imputed to modal values for categorical covariates and median values for ordinal and continuous characteristics to retain as many cases as possible. Values for missing baseline COVID-19 vaccination intention were taken from the earliest available follow-up survey where vaccination intention was reported (typically within a month of the baseline survey and always before the availability of vaccines). The impact of the imputation on results was assessed in the primary analysis.

## 3. Results

### 3.1. Baseline Vaccination Intention

When asked on the baseline survey whether they planned to receive a COVID-19 vaccine when it became available, 761 (62.2%) participants said ‘Yes’, 372 (30.4%) were ‘Unsure’ and 90 (7.4%) said ‘No.’ Table 2 describes the characteristics of the analysis population and characteristics by baseline vaccination intention groups. The overall population mirrors the characteristics of the overall C3PI cohort, while characteristics by participant baseline vaccination intention groups (No/Unsure/Yes) differed on all characteristics. [33] Generally, participants in the ‘No’ and ‘Yes’ intention groups tended to be the most different. Compared with participants who stated they intended to receive a COVID-19 vaccine, those who stated they did not intend to receive a COVID-19 vaccine at baseline were younger (58.0 vs. 62.8 years old, *p* ≤ 0.0001), more often female (80.0% vs. 63.5%, *p* ≤ 0.0001) or a minority race (minority race: 24.5% vs. 7.2%, *p* ≤ 0.0001) or of Hispanic ethnicity (5.6% vs. 3.0%, *p* ≤ 0.0027), had a lower education level (some college or less: 35.6% vs. 25.2%, *p* ≤ 0.0001) and annual household income (<$50,000: 27.8% vs. 14.6%, *p* ≤ 0.0001), and higher BMI (30.7 vs. 28.2, *p* = 0.0003).

A total of 1130 (92.4%) participants were vaccinated during the study. Of those vaccinated, 745 (65.9%) were part of the ‘Yes’ baseline COVID-19 vaccination intention group and 334 (29.6%) and 51 (4.5%) were part of the ‘Unsure’ and ‘No’ baseline vaccination intention groups, respectively. Overall, 97.9% of those who stated they would receive a COVID-19 vaccine at baseline received a COVID-19 vaccine during their participation in the study. In comparison, 89.9% and 56.7% of those who answered ‘Unsure’ and ‘No’ received a COVID-19 vaccine during the study (*p* < 0.0001).

### 3.2. Vaccination Promptness

A total of 314 (25.7%) participants received vaccines before their estimated earliest eligibility date, while 816 (66.7%) were vaccinated after. Characteristics associated with participants who were vaccinated before eligibility included participation in a COVID-19 vaccine clinical trial, older age, male sex, and an increased likelihood of stating ‘Yes’ on the baseline vaccination intention survey (Table S1).

**Figure 2:**
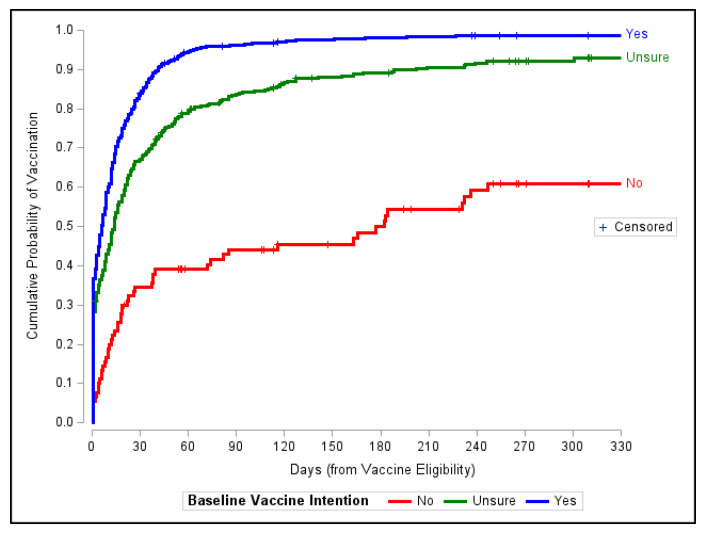
Cumulative probabilities of vaccination based on Kaplan–Meier survival curves for vaccination promptness as a function of baseline vaccination intention (No/Unsure/Yes).

Initial Kaplan–Meier survival analyses showed strong relationships between time to vaccination (i.e., vaccination promptness) and baseline vaccination intentions (Figure 2). Among those who received their COVID-19 vaccine during their study participation (1130 participants, 92.4%), the median time to vaccination following their eligibility date was 9 days (IQR: 8–11). Those in the ‘Yes’ baseline vaccination intention group received their COVID-19 vaccine faster (median 6 days (IQR: 5–8)) than those in the ‘Unsure’ (median 14 days (IQR: 11–16)) and ‘No’ (median 182 days (IQR: 74 –>343 days)) baseline vaccination intention groups. By pairwise log-rank tests, all vaccination intention groups statistically differed in vaccination promptness (Yes vs. No: χ^2^(1) = 167.20; *p* ≤ 0.0001; Yes vs. Unsure: χ^2^(1) = 68.62; *p* ≤ 0.0001; No vs. Unsure: χ^2^(1) = 5.13; *p* ≤ 0.02).

### 3.3. Factors Affecting Vaccination Promptness

In bivariate analyses, the set of candidate predictors were reduced to age, dichotomized race (white/minority), sex, Hispanic ethnicity, dichotomized education level (less than college graduate/college graduate or higher), dichotomized household income (less than $50 k/more than $50 k), dichotomized BMI (overweight or less/obese or greater), and baseline vaccination intention (No/Unsure/Yes). No results were sensitive to the inclusion of imputed values, nor did the ‘missing at random’ assumption appear to be violated.

Multivariable Cox proportional hazard models were used to identify the strongest subset of predictors and interactions. Among the interactions, only the one between race and vaccination intention was statistically significant. The proportional hazards assumption failed to hold for baseline vaccination intention, which was addressed by including an interaction between baseline vaccination intention and log time to vaccination. Martingale residuals suggested a quadratic relationship between age and time to vaccination, so both age and age^2^ terms were included in the model. The resulting full Cox proportional hazards model is shown in Table S2. The final model, which drops the non-significant BMI and interaction terms, is shown in Table 3. After controlling for covariates (sex, minority race, and Hispanic ethnicity), a lower education level (some college or less) was associated with a 20% lower (HR = 0.80 [0.69–0.92]) likelihood of vaccination relative to those with higher education. Lower levels of household income (less than $50 k vs. >$50 k) were associated with a 16% lower (HR = 0.84 [0.72–0.98]) vaccination likelihood over time.

**Figure 3:**
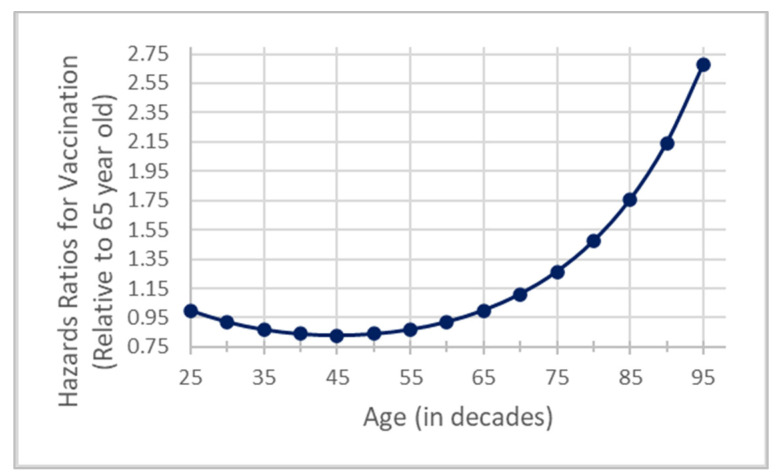
Quadratic age effect on vaccination hazard ratios calculated relative to a 65-year-old.

Figure 3 shows the quadratic relationship between vaccination probability and baseline age. For example, compared with a 65-year-old, a 45-year-old had a 17% lower vaccination likelihood (HR = 0.83 [0.74–0.93]), while an 85-year-old had a 76% higher vaccination likelihood (HR = 1.76 [1.37–2.25]).

**Figure 4:**
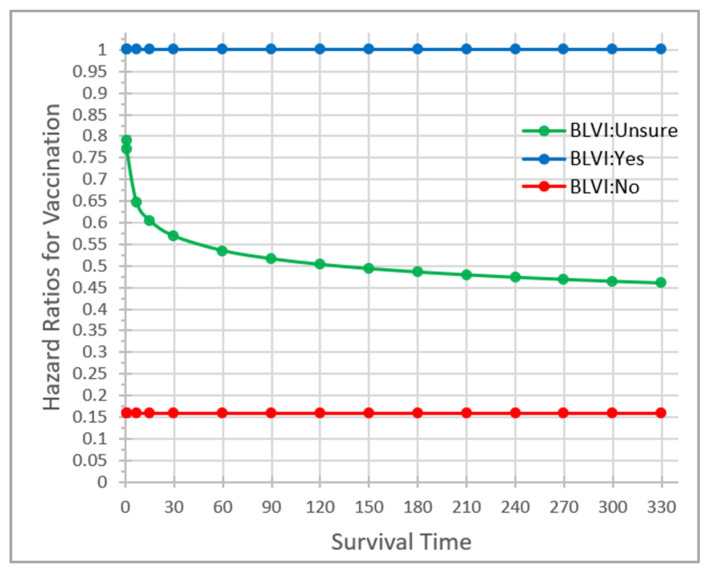
Changing probability of vaccination over time by baseline COVID-19 vaccination intention. Vaccination = ’Yes’ is the reference group.

Relative to those who, at baseline, stated they intended to receive the COVID-19 vaccine, participants who expressed their intention not to be vaccinated had a consistent 84% lower (HR = 0.16 [0.11–0.23]) probability of becoming vaccinated over time since eligibility. Additionally, on average, those who were ‘Unsure’ had a 23% lower probability of vaccination (HR = 0.77 [0.63–0.94]); however, their vaccination likelihoods varied over time since eligibility. Figure 4 depicts the interaction between vaccination probability and time since eligibility for participants who were ‘Unsure’ of their vaccination intention at baseline. In the figure, the flat line at a hazard ratio of 0.16 (0.11–0.23) represents the constant vaccination likelihood over time for participants with a ‘No’ intention of vaccination at baseline. The monotonically decreasing curve describes vaccination likelihoods over time since eligibility for participants who were ‘Unsure’ of their vaccination intentions at baseline, showing that the longer (from eligibility) participants remained unsure about getting vaccinated, the less likely they were to get vaccinated.

**Figure 5:**
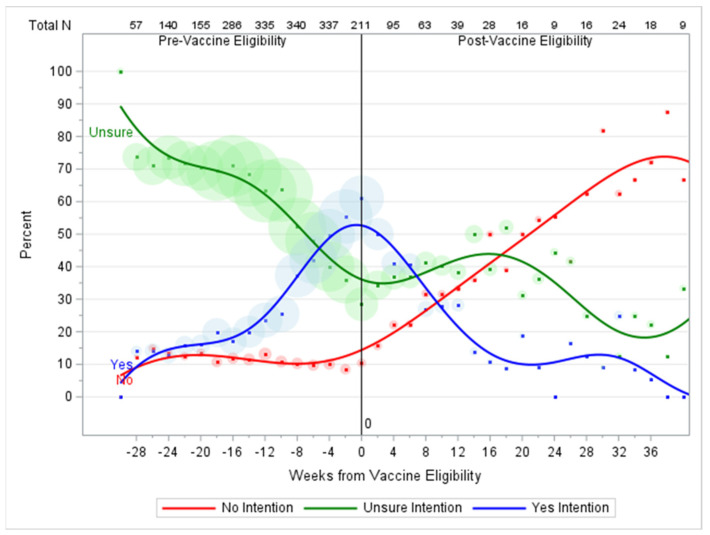
Trends in vaccination intention over time for study participants who expressed ‘Unsure’ COVID-19 vaccination intention at baseline (N = 372). The *y*-axis is the percentage of all cases with data in a week who expressed each vaccination intention. The area of the dots is proportional to the count of participants in a week who expressed each intention, with the smallest dots representing counts of 0-3 and the largest representing up to 225. The numbers at the top of the graph are total participants with data at selected weeks.

Figure 5 shows the patterns of changing vaccination intention among the 372 participants who expressed ‘Unsure’ vaccination intentions at baseline. The proportion of the unvaccinated members of this group expressing uncertainty about vaccination declined over time, eventually stabilizing around 25% of the unvaccinated population by about 28 weeks post-eligibility. Early, this decline was driven by an increase in the proportion of ‘Yes’ intentions and later by increases in ‘No’ intentions. The fraction of ‘Yes’ intentions started at 0%, increased until eligibility, then declined again to 0% as participants who shifted to ‘Yes’ received their vaccinations. The fraction of ‘No’ intentions started at 0% and stayed low until eligibility. They then increased during the post-eligibility period, reflecting a small but proportionally increasing group who hardened from ‘Unsure’ to ‘No’ after they became eligible for vaccination.

**Figure 6:**
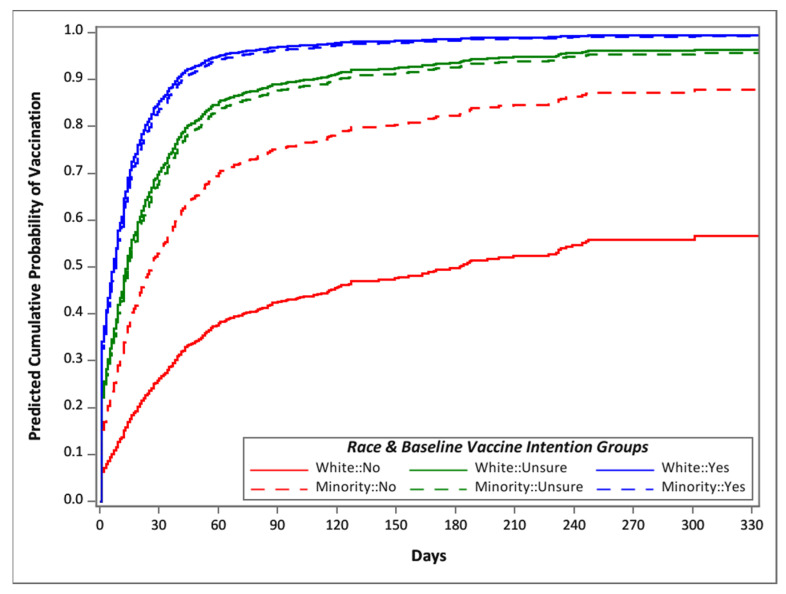
Predicted cumulative probabilities of vaccination based on Cox model survival curves for time to vaccination after eligibility. Separate curves are shown by baseline COVID-19 vaccination intention and race. All other variables from Table 3 are adjusted to reference levels.

Minority participants who expressed baseline vaccination intentions of ‘Yes’ or ‘Unsure’ had approximately the same likelihood of vaccination as white participants who expressed a baseline vaccination intention of ‘Yes’ or ‘Unsure’ (6% lower likelihood, HR = 0.94 [0.77, 1.16]). However, minority participants who expressed a baseline vaccination intention of ‘No’ (HR = 0.40 [0.25, 0.64]) had about 2.5 times higher likelihood of vaccination than white participants who expressed a baseline vaccination intention of ‘No’ (HR = 0.16 [0.11, 0.23]). Thus, although both white and minority participants who expressed ‘No’ intention to be vaccinated at baseline had substantially lower likelihoods of vaccination than those who voiced baseline vaccination intentions of ‘Yes’ or ‘Unsure’, minority participants who said ‘No’ were more likely to get vaccinated than their white counterparts. Figure 6 depicts predicted cumulative probabilities of vaccination (based on adjusted survival curves) broken down by minority and baseline vaccination intention groups.

### 3.4. Reasons for Vaccination Hesitancy

An amount of 57 (98.3%) of the 58 participants who remained unvaccinated during Phase 2 of the study provided reasons for their vaccination hesitancy on surveys sent to participants on 24 May 2021. Participants selected a mean of 2.07 (SD = 1.24) reasons for their COVID-19 vaccination hesitancy for cycle 1. Selected reasons included concerns with vaccine safety (n = 26, 45.6%), concerns with vaccine side effects (n = 26, 45.6%), concerns with vaccine effectiveness (n = 17, 29.8%), not concerned about becoming infected and becoming ill with COVID-19 (n = 15, 26.3%), concerns based on the experience of a friend or family member (n = 10, 17.5%), religious or other exemption (n = 8, 14.0%), and fear of needles (n = 1, 1.8%). Fifteen participants (26.3%) also selected “Other” for vaccination hesitancy. When asked to specify their other reason, most participants’ answers revolved around previously having COVID-19 (n = 8, 53.3%), medical advice/medical reasons (n = 3, 20.0%), or misinformation/government concerns (n = 2, 13.3%). No participant selected “it would be hard for me due to transportation, other costs or burden on me” or “I have not been able to schedule an appointment yet”.

Fifty-three (91.4%) of the 58 participants who remained unvaccinated at the end of Phase 2 of the study continued their involvement until the end of the study (week of 22 November 2021). The remaining five participants ended their participation in May, June, July, September, and October of 2021. At the time of their last survey, participants selected a mean of 2.38 (SD = 1.54) reasons why they had not received the COVID-19 vaccine. The most common reasons were concerns about vaccine safety (n = 33, 56.9%), side effects of vaccination (n = 28, 43.3%), and vaccine effectiveness (n = 25, 43.1%). Eleven participants (19.0%) also selected other reasons for their vaccination hesitancy; these answers revolved around previously having COVID-19 (n = 6, 54.5%), medical advice/medical reasons (n = 3, 27.3%), or misinformation/government concerns (n = 2, 18.2%). No participant selected the “I have not been able to schedule an appointment yet,” “I am afraid of needles,” or “I feel it would be hard for me due to transportation, other costs or burden on me” options.

## 4. Discussion

Since the start of the COVID-19 pandemic, COVID-19 has caused a widespread global impact on all aspects of life, including global health. Due to these devastating effects, solutions to stop the spread of COVID-19 were rapidly developed, including vaccines. After vaccines were approved, the next obstacle became getting people vaccinated to reach herd immunity. Vaccination hesitancy continues to impede herd immunity and has been recognized as a threat to immunization programs worldwide [18,19,20]. In addition, prompt vaccination after eligibility reduces unprotected exposure time to COVID-19. We evaluated vaccination promptness and found key factors associated with COVID-19 vaccination promptness, including early vaccination intention (before availability of COVID-19 vaccines). We further identified participant-reported reasons for vaccination hesitancy among those who remained unvaccinated after vaccines were made widely available to adults in the United States.

In our study, most participants (92.4%) received a COVID-19 vaccine. Participants with a baseline vaccination intention of ‘Yes’ and older participants more rapidly received a COVID-19 after becoming eligible. Those with lower education levels, lower household incomes, and who expressed a baseline vaccination intention of ‘No’ took longer to receive a COVID-19 vaccine after eligibility. While minority populations in North Carolina are more hesitant than white populations in North Carolina [43], our study found minority participants who expressed a baseline vaccination intention of ‘No’ were more likely over time to get a COVID-19 vaccine than white participants with a baseline vaccination intention of ‘No.’

To our knowledge, no other study has looked at characteristics associated with the rapidity of becoming vaccinated after becoming eligible; however, other studies have investigated vaccine uptake and intention related to COVID-19. In the United States, vaccine uptake has been found to be associated with a person’s intention to be vaccinated in various populations [49,50]. Additionally, one study found that the COVID-19 vaccine intentions of Black individuals from the United States were initially comparable to White individuals from the United States; however, the Black individuals experienced a more rapid increase in belief that the vaccines were necessary for protection and vaccine intention over time [51]. One study from North Carolina found higher rates of vaccine uptake among older individuals (97.2% in those 70+), those with no previous COVID-19 diagnosis (92.7% vs. 81.9%), and those who lived in urban settings (urban: 94.3%; rural: 90.2%; suburban: 88.4%) [52]. Additionally, Enwezor et al. found an 88.9% COVID-19 vaccine uptake among Black participants who had previously expressed negative pre-vaccination attitudes. However, none of these studies examined the promptness of vaccination.

We also examined vaccination hesitancy among the small cohort of study participants who remained unvaccinated after vaccines were widely indicated. Reasons for vaccination hesitancy are very nuanced and are typically driven by many interconnecting social and political factors [18,53]. Our sample size (N = 58) was too small to quantitatively examine sociodemographic and political influences on vaccination hesitancy. However, we did summarize reasons participants selected for not being vaccinated. The most common reasons for not receiving a vaccine included concerns with vaccine safety, side effects of vaccination, and vaccine effectiveness. Less frequently, participants cited previously having COVID-19, medical advice/medical reasons, or misinformation/government concerns as their reasons for remaining unvaccinated. Notably absent were religious objections to vaccination. The findings of our study are similar to those of other studies. According to the 2021 United States Household Pulse Survey, the most common reasons North Carolinians did not want to receive the COVID-19 vaccine were not trusting the vaccine, not trusting the government, and not believing they needed it [21]. Other studies in North Carolina and the United States have also found the lack of trust in COVID-19 vaccines’ safety, effectiveness, and necessity among the top reasons reported for not wanting to receive the vaccine or not vaccinating their child [53,54,55,56,57,58].COVID–19 vaccines were created at a record-breaking time, with large pharmaceutical companies racing to be the first to have a vaccine. This may have led to skepticism about the vaccine and the priorities of pharmaceutical companies [18,54,58]. There is also a staggering amount of information and misinformation about COVID-19 and COVID-19 vaccines on the internet and in the media, which could lead individuals to struggle with determining which piece/s of information to believe and act upon [18,53].

### 4.1. Strengths and Limitations

The study’s strengths include (1) the use of a large community-based population within North Carolina and (2) the use of data from repeated survey waves that enabled the examination of COVID-19 vaccination over time. Vaccination promptness was a function of key characteristics, such as baseline vaccination intention, race, and age, and was controlled for other participant factors to reduce confounding. This study is one of the first to examine time to vaccination relative to eligibility rather than vaccine availability. The present study also has limitations. The cohort was regionally based, had limited diversity (69.3% women and 87.4% white race), and was highly educated (70.3% college graduate or higher), which may limit generalizability to other populations. Additionally, determining the earliest vaccine eligibility dates was imprecise for a small but non-negligible fraction of the sample. Age and medical condition-based eligibility groups were well-determined and unambiguous. However, classifying individuals into employment-based vaccine priority groups other than healthcare workers was more difficult as our survey questions were designed before the NCDHHS employment-based vaccine eligibility group criteria were available.

### 4.2. Implications for the Future

These study results could help policymakers and healthcare professionals address populations at risk for delaying vaccination or not being vaccinated. Our research showed that targeting those expressing ‘No’ or ‘Uncertain’ vaccination intention may be particularly fruitful and highlighted characteristics that, in general, may help to identify these groups. In particular, individuals who are uncertain seem to become more definite in their attitudes over time, and the longer they remain unvaccinated, the less likely they will be vaccinated. Thus, a crucial intervention for individuals with vaccination ambivalence may lie in early persuasion to vaccination. People who express hesitation about the COVID-19 vaccines are more likely to be reluctant towards other vaccines, like influenza or childhood vaccines [25,59,60,61,62,63,64], so these results may also apply to other vaccines. More detailed examinations of patterns of vaccination attitudes may reveal additional avenues or potential intervention targets for encouraging early or eventual vaccination—especially among those initially ‘Unsure’ of their intentions.

## 5. Conclusions

Failure to vaccinate is recognized as a threat to infectious disease control programs worldwide. Further, with a highly transmissible illness like COVID-19, the promptness of vaccination may play a major role in limiting illness and death. Several sociodemographic factors and vaccination intention appear important to vaccination promptness. Additionally, concerns about vaccine side effects, safety and effectiveness, trust in vaccination, and need for vaccination are addressable factors in vaccination hesitancy. These findings could inform targeted vaccination campaigns in future vaccination rollouts that prioritize groups most likely to delay and that address vaccine concerns to improve vaccination promptness and reduce hesitancy.

## Figures and Tables

**Table 1:** NC vaccination priority groups, vaccination eligibility dates, and definitions, including a fraction of the analysis sample whose earliest vaccination eligibility dates were determined by qualification under each priority group criterion.

Vaccination Group	Vaccination Eligibility Date	Definition	Analysis Sample n (%)
Priority Group 1	12/14/2020	Healthcare workers, residents and staff of long-term care facilities	145 (11.9%)
Priority Group 2	1/18/2021	Older adults (age 65+)	531 (43.4%)
Priority Group 3a	2/24/2021	Primary, secondary, and preschool teachers and other school staff	68 (5.6%)
Priority Group 3b	3/3/2021	Other frontline essential workers	141 (11.5%)
Priority Group 4a	3/17/2021	People with high-risk medical conditions; People living in close group settings like homeless shelters, jails, or prisons	289 (23.6%)
Priority Group 4b	3/30/2021	Other essential workers; other people living in group settings like dormitories or fraternity houses	9 (0.7%)
All adults (Ages 16+)	4/7/2021	Everyone 16 years of age or older	40 (3.3%)

**Table 2:** Baseline Characteristics of analysis population overall and by baseline vaccination intention group (No/Unsure/Yes).

Variables	Analysis Population (n = 1223)	BL Vax Intent = ‘NO’ (n = 90)	BL Vax Intent = ‘UNSURE’ (n = 372)	BL Vax Intent = ‘YES’ (n = 761)	*p*-Value ^+^
Baseline Age in years					0.0001
Mean ± SD	61.2 ± 12.1	58.0 ± 11.3	58.7 ± 12.3	62.8 ± 11.8	
Min–Max	26.0–90	26.0–81.0	26.0–87.0	27.0–98.0	
Sex					0.0001
Female	848 (69.3%)	72 (80.0%)	293 (78.8%)	483 (63.5%)	
Male	375 (30.7%)	18 (20.0%)	79 (21.2%)	278 (36.5%)	
Race					0.0001
White	1069 (87.4%)	68 (75.6%)	295 (79.3%)	706 (92.8%)	
Black	102 (8.3%)	15 (16.7%)	51 (13.7%)	36 (4.7%)	
Other	52 (4.3%)	7 (7.8%)	26 (7.0%)	19 (2.5%)	
Hispanic Ethnicity					0.0027
Non-Hispanic	1167 (95.4%)	85 (94.4%)	344 (92.5%)	738 (97.0%)	
Hispanic	56 (4.6%)	5 (5.6%)	28 (7.5%)	23 (3.0%)	
Education Level					0.0001
HS or less	85 (7.0%)	7 (7.8%)	43 (11.6%)	35 (4.6%)	
Some College	278 (22.7%)	25 (27.8%)	96 (25.8%)	157 (20.6%)	
College Grad	480 (39.2%)	40 (44.4%)	136 (36.6%)	304 (39.9%)	
Graduate School	380 (31.1%)	18 (20.0%)	97 (26.1%)	265 (34.8%)	
Household Income					0.0001
<30,000	96 (7.8%)	7 (7.8%)	44 (11.8%)	45 (5.9%)	
30,000–49,999	144 (11.8%)	18 (20.0%)	60 (16.1%)	66 (8.7%)	
50,000–74,999	195 (15.9%)	15 (16.7%)	52 (14.0%)	128 (16.8%)	
75,000–89,999	213 (17.4%)	14 (15.6%)	69 (18.5%)	130 (17.1%)	
90,000+	575 (47.0%)	36 (40.0%)	147 (39.5%)	392 (51.5%)	
BMI					0.0003
Mean ± SD	28.6 ± 6.1	30.7 ± 7.3	29.0 ± 5.9	28.2 ± 5.9	
Min–Max	16.0–62.0	17.3–62.0	16.0–53.7	16.0–54.1	
BMI Group					0.0273
Underweight (BMI < 18.5)	15 (1.2%)	1 (1.1%)	6 (1.6%)	8 (1.1%)	
Healthy (18.5 ≤ BMI < 25)	327 (26.7%)	16 (17.8%)	86 (23.1%)	225 (29.6%)	
Overweight (25 ≤ BMI < 30)	467 (38.2%)	30 (33.3%)	146 (39.2%)	291 (38.2%)	
Obese (30 ≤ BMI < 40)	350 (28.6%)	34 (37.8%)	116 (31.2%)	200 (26.3%)	
Severely Obese (BMI ≥ 40)	64 (5.2%)	9 (10.0%)	18 (4.8%)	37 (4.9%)	
Current Smoker					0.0353
Yes	39 (3.2%)	3 (3.3%)	19 (5.1%)	17 (2.2%)	
No	1184 (96.8%)	87 (96.7%)	353 (94.9%)	744 (97.8%)	
Received a COVID-19 vaccine during the study period					<0.0001
Yes	1130 (92.4%)	51 (56.7%)	334 (89.8%)	745 (97.9%)	
No	93 (7.6%)	39 (43.3%)	38 (10.2%)	16 (2.1%)	

Note: SD = standard deviation; Min = minimum; Max = maximum; BMI = body mass index; + *p*-values are based on ANOVAs (2 degrees of freedom tests for distributional differences between the BVI groups) for continuous characteristics and likelihood ratio χ^2^ tests of general association for categorical characteristics.

**Table 3:** Final reduced Cox proportional hazards model for vaccination promptness following eligibility. Reference categories (not shown) included female, non-minority, non-Hispanic, education ≥ college graduate, income ≥ $50,000, and baseline vaccination intention = ’Yes.’

Characteristic	Param Est. (SE)	Hazard Ratio (95% CI)	Joint Test *p*-Value	Pairwise Comparison *p*-Value
Age at baseline (in decades, centered at 65)	0.19 (0.03)	1.21 (1.13–1.29)	**0.0001**	
Age^2^ at baseline (in decades, centered at 65)	0.05 (0.02)	1.05 (1.01–1.06)	**0.0066**	
Male	−0.06 (0.07)	0.95 (0.83–1.08)	0.3947	
Minority Race	−0.06 (0.11)	0.95 (0.77–1.16)	0.5900	
Hispanic	0.18 (0.15)	1.20 (0.88–1.62)	0.2466	
Some College or less	−0.23 (0.07)	0.80 (0.69–0.92)	**0.0014**	
Income < $50,000	−0.17 (0.08)	0.84 (0.72–0.98)	**0.0312**	
BL Vaccination Intention (‘Yes’ = ref)			**0.0001**	
No	−1.84 (0.19)	0.16 (0.11–0.23)		**0.0001**
Unsure	−0.26 (0.10)	0.77 (0.63–0.94)		**0.0088**
Minority Race XBL Vaccination Intention (BVI)				
Minority: Yes X BVI = ‘Unsure’	0.98 (0.31)	2.67 (1.46–4.88)		**0.0014**
BL Vaccination Intention X Time to Vaccination			0.0535	
BVI = ’Unsure’ X Time to Vaccination	−0.09 (0.04)	0.92 (0.85–0.99)		**0.0203**

Note: SE = standard error; CI = confidence interval; BL = baseline; BVI = baseline vaccination intention.

## Data Availability

The data presented in this study are available on request from L Kristin Newby at kristin.newby@duke.edu.

## Supplementary Materials

The following supporting information can be downloaded at https://www.mdpi.com/article/10.3390/vaccines11111639/s1. Table S1: Characteristics of sample members who were vaccinated before their estimated eligibility date vs. those vaccinated after their estimated eligibility date; Table S2: Results from full Cox proportional hazards model.
