# Supplementary material for: Factors Associated with COVID-19 Vaccination Promptness after Eligibility in a North Carolina Longitudinal Cohort Study
Source: Vaccines (Basel). 2023 Oct 26;11(11):1639. doi: 10.3390/vaccines11111639 (PMC10674190; doi:10.3390/vaccines11111639)
Supplement: Supplementary file 1 [file vaccines-11-01639-s001.zip › vaccines-2645542-supplementary.pdf]

Table S1: Results from full Cox proportional hazards model.

Characteristic	Param Est. (Std. Err.)	Hazard Ratio (95% CI)	Joint Test p-value	Pairwise Comparison p-value
Baseline Age (in decades, centered at age 65)	.18 (.04)	1.20 (1.12 - 1.28)	**.0001**	
Baseline Age^2	.04 (.02)	1.05 (1.01 - 1.08)	**.0093**	
Sex (Male)	-.05 (.07)	.95 (.83 - 1.08)	.4316	
Minority Race (Yes)	-.17 (.15)	.84 (.63 - 1.13)	.2547	
Hispanic (Yes)	.17 (.15)	1.19 (.88 - 1.61)	.2603	
Educ2 (Some College or less)	-.22 (.07)	.80 (.70 - .92)	**.0021**	
Income2 (< $50,000)	-.16 (.08)	.86 (.73 - 1.00)	**.0564**	
BMI2 (Obese/Sev.Obese)	-.08 (.07)	.92 (.81 - 1.05)	.2171	
BL Vaccine Intention			**.0001**	
No	-1.94 (.32)	.14 (.08 - .27)		**.0001**
Unsure	-.29 (.10)	.75 (.61 - .91)		**.0046**
Minority Race*BL Vaccine Intention			**.0034**	
Minority:Yes*VI:No	1.11 (.33)	3.02 (1.59 - 5.75)		**. 0007**
Minority:Yes*VI:Unsure	.23 (.20)	1.26 (.85 - 1.87)		. 2417
BL Vaccine Intention*Survival Time			**.0535**	
VI:No*Survival Time	.03 (.09)	1.03 (.87 - 1.23)		.6994
VI:Unsure*Survival Time	-.09 (.04)	.92 (.85 - .99)		**.0235**

Table S2. Characteristics of sample members who were vaccinated before their estimated eligibility date vs. those vaccinated after their estimated eligibility date.

Variables	Total Vaccinated Sample (N=1130)	Vaccinated Before Eligibility (N=314)	Vaccinated After Eligibility (N=816)	p-value*
**COVID vaccine trial participant**				<.0001
No	1065 (94.2%)	282 (89.8%)	783 (96.0%)	
Yes	65 (5.8%)	32 (10.2%)	33 (4.0%)	
**Baseline Age in years**				0.0090
Mean±SD	61.6±12.0	63.1±12.6	61.0±11.8	
Min–Max	27.0–98.0	30.0–98.0	27.0–94.0	
**Sex**				0.0200
Female	785 (69.5%)	202 (64.3%)	583 (71.4%)	
Male	345 (30.5%)	112 (35.7%)	233 (28.6%)	
**Race**				0.1987
White	981 (87.5%)	277 (89.6%)	704 (86.7%)	
Black	92 (8.2%)	18 (5.8%)	74 (9.1%)	
Other	48 (4.3%)	14 (4.5%)	34 (4.2%)	
missing	*9 (0.8%)*	*5 (1.6%)*	*4 (0.5%)*	
**Hispanic Ethnicity**				0.8673
Non-Hispanic	1069 (95.4%)	297 (95.2%)	772 (95.4%)	
Hispanic	52 (4.6%)	15 (4.8%)	37 (4.6%)	
missing	*9 (0.8%)*	*2 (0.6%)*	*7 (0.9%)*	
**Education Level**				0.1778
HS or less	72 (6.4%)	12 (3.9%)	60 (7.4%)	
Some College	235 (21.0%)	70 (22.7%)	165 (20.4%)	
College Grad	442 (39.5%)	124 (40.3%)	318 (39.3%)	
Graduate School+	369 (33.0%)	102 (33.1%)	267 (33.0%)	
missing	*12 (1.1%)*	*6 (1.9%)*	*6 (0.7%)*	
**Household Income**				0.0809
< 30,000	84 (7.9%)	13 (4.4%)	71 (9.2%)	
30,000-49,999	129 (12.1%)	31 (10.5%)	98 (12.7%)	
50,000-74,999	182 (17.1%)	54 (18.4%)	128 (16.6%)	
75,000-89,999	133 (12.5%)	39 (13.3%)	94 (12.2%)	
90,000+	537 (50.4%)	157 (53.4%)	380 (49.3%)	

Variables	Total Vaccinated Sample (N=1130)	Vaccinated Before Eligibility (N=314)	Vaccinated After Eligibility (N=816)	p-value*
missing	*65 (5.8%)*	*20 (6.4%)*	*45 (5.5%)*	
**BMI**				0.1305
Mean±SD	28.5±6.0	28.1±5.9	28.7±6.1	
Min–Max	16.0–54.1	17.5–54.1	16.0–53.7	
missing (%)	*28 (2.5%)*	*11 (3.5%)*	*17 (2.1%)*	
**BMI Group**				0.4639
Underweight (BMI < 18.5)	13 (1.2%)	4 (1.3%)	9 (1.1%)	
Healthy (18.5 ≤ BMI < 25)	309 (28.0%)	97 (32.0%)	212 (26.5%)	
Overweight (25 ≤ BMI < 30)	411 (37.3%)	109 (36.0%)	302 (37.8%)	
Obese (30 ≤ BMI < 40)	313 (28.4%)	79 (26.1%)	234 (29.3%)	
Severely Obese (BMI ≥ 40)	56 (5.1%)	14 (4.6%)	42 (5.3%)	
missing	*28 (2.5%)*	*11 (3.5%)*	*17 (2.1%)*	
**Current Smoker**				0.1116
No	1088 (96.7%)	306 (98.1%)	782 (96.2%)	
Yes	37 (3.3%)	6 (1.9%)	31 (3.8%)	
missing	*5 (0.4%)*	*2 (0.6%)*	*3 (0.4%)*	
**Baseline COVID Vaccine Intention**				0.0003
No	49 (4.4%)	3 (1.0%)	46 (5.7%)	
Maybe/Unsure	328 (29.7%)	79 (26.0%)	249 (31.0%)	
Yes	729 (65.9%)	222 (73.0%)	507 (63.2%)	
missing	*24 (2.1%)*	*10 (3.2%)*	*14 (1.7%)*	

***** p-values are based on t-tests with Satterwaite-adjusted standard errors for continuous characteristics and likelihood ratio χ^2 tests of general association for categorical characteristics. Missing values are shown as a fraction of the group sample size, but not included in the statistical tests.
